# Lineage Reprogramming of Effector Regulatory T Cells in Cancer

**DOI:** 10.3389/fimmu.2021.717421

**Published:** 2021-07-28

**Authors:** Michael L. Dixon, Jonathan D. Leavenworth, Jianmei W. Leavenworth

**Affiliations:** ^1^Department of Neurosurgery, University of Alabama at Birmingham, Birmingham, AL, United States; ^2^Graduate Biomedical Sciences Program, University of Alabama at Birmingham, Birmingham, AL, United States; ^3^Department of Dermatology, University of Alabama at Birmingham, Birmingham, AL, United States; ^4^Department of Microbiology, University of Alabama at Birmingham, Birmingham, AL, United States; ^5^The O’Neal Comprehensive Cancer Center, University of Alabama at Birmingham, Birmingham, AL, United States

**Keywords:** anti-tumor immunity, effector regulatory T cells, follicular regulatory T cells, Foxp3, Treg lineage stability, humoral antibody response

## Abstract

Regulatory T-cells (Tregs) are important for maintaining self-tolerance and tissue homeostasis. The functional plasticity of Tregs is a key feature of this lineage, as it allows them to adapt to different microenvironments, adopt transcriptional programs reflective of their environments and tailor their suppressive capacity in a context-dependent fashion. Tregs, particularly effector Tregs (eTregs), are abundant in many types of tumors. However, the functional and transcriptional plasticity of eTregs in tumors remain largely to be explored. Although depletion or inhibition of systemic Tregs can enhance anti-tumor responses, autoimmune sequelae have diminished the enthusiasm for such approaches. A more effective approach should specifically target intratumoral Tregs or subvert local Treg-mediated suppression. This mini-review will discuss the reported mechanisms by which the stability and suppressive function of tumoral Tregs are modulated, with the focus on eTregs and a subset of eTregs, follicular regulatory T (T_FR_) cells, and how to harness this knowledge for the future development of new effective cancer immunotherapies that selectively target the tumor local response while sparing the systemic side effects.

## Introduction

An effective immune system must be capable of maintaining self-tolerance while generating robust responses to foreign antigens. Tregs are important components participating in such immune regulation (1, 2). In both human and mice, Tregs are characterized by their high expression of both the IL-2 receptor α-chain (CD25) and the transcription factor Foxp3, which are essential for their development, suppressive activity and stability (3–8). Foxp3^+^ Tregs comprise both central Treg (cTreg) and eTreg subsets (9, 10). Accumulation of Tregs, particularly eTregs, within the tumor represents a major obstacle to the development of effective anti-tumor immunity (11–13). The frequency of Tregs among tumor-infiltrating lymphocytes (TIL) is often associated with poor prognosis of patients with many types of cancer (14), although Tregs can also be beneficial during early stages of inflammation-related cancers, such as colorectal cancer, and correlate with better prognosis (15–18). Substantial reviews have discussed the homeostatic regulation of Tregs and their suppressive function, including the most recent one centering on tumoral Tregs (19). This review will cover Treg stability with a focus on eTregs and T_FR_ cells, and how their stability affects cancer progression and how it can be targeted for therapy.

## Treg and eTreg Biology

Tregs mediate suppression through various mechanisms including obstructing CD80/CD86 co-stimulation *via* the surface receptor CTLA-4, limiting IL-2 availability for effector T-cells (Teff) and secreting inhibitory molecules IL-10, IL-35 or TGF-β (20). However, Tregs are phenotypically and functionally diverse. Based on the developmental origin, Tregs are defined as either thymic or peripheral Tregs. Thymic Tregs (tTregs) begin as CD4 single positive thymocytes with TCRs displaying high affinity for self-antigens. Peripheral Tregs (pTregs) develop from naïve CD4^+^ T-cells in the periphery that experience antigen and receive specific environmental stimuli, such as TGF-β and IL-2 (21, 22). Although the definitive markers distinguishing tTregs from pTregs remain obscure, all Tregs in the periphery reside in multiple lymphoid and non-lymphoid tissues to maintain tolerance or suppress ongoing inflammatory responses. In the circulation and lymphoid organs, the majority of Tregs that express the homing receptors CD62L and CCR7, but low level of CD44, are cTregs and are largely IL-2-dependent (9). In contrast, a large population of Tregs in the non-lymphoid tissues that have a CD44^hi^CD62L^lo^CCR7^lo^ surface phenotype resembling activated or effector conventional T-cells are eTregs (9, 23). In the presence of TCR, CD28 and IL-2 signaling, cTregs differentiate into eTregs accompanying the upregulation of IRF4 and Blimp1 (23, 24). eTregs can further undergo stimulus-specific differentiation that is regulated by signals and transcription factors typically associated with the differentiation of conventional T-helper (T_H_) cells. This polarization allows Tregs to regulate specific immune responses mediated by their analogous effector CD4^+^ T-cells in addition to their generic suppressive capacity (23). In addition to the high level of CD44, eTregs express effector markers, including ICOS and GITR (10, 24). Analogous subsets also exist for human Tregs, including resting FOXP3^lo^CD45RA^+^ and effector FOXP3^hi^CD45RA^–^ suppressive subsets, while FOXP3^lo^CD45RA^–^ cells are non-suppressive cytokine-secreting subsets (25). Importantly, CD15s has been identified as a biomarker for most suppressive human FOXP3^hi^ eTregs (26). Although eTregs are predominantly found in non-lymphoid tissues, B-cell follicles in the lymphoid or lymphoid-like organs contain a subset of eTreg, known as T_FR_ cells, which are responsible for regulating the follicular helper T (T_FH_)–B-cell interaction in the germinal center (GC), and thus the production of high-affinity antibody (27–30).

## T_FR_ cell Biology

T_FR_ cells share many features with T_FH_ cells, but they express Foxp3 and belong to eTregs. Like T_FH_ cells, T_FR_ cells express high levels of PD-1 and CXCR5, which allows them to traffic to B-cell follicles following the chemokine CXCL13 gradients (27–30). Both T_FR_ and T_FH_ cells require ICOS and CD28 signaling for their development and maintenance and are dependent of antigen presenting cells and B-cells in the GC (27–31). T_FH_ and T_FR_ cells express high levels of Bcl6, however, unlike T_FH_ cells, T_FR_ cells also co-express Blimp1, which antagonizes Bcl6. While Bcl6 is critical for the development of T_FR_ cells as depletion of Bcl6 results in an almost complete loss of T_FR_ cells, Blimp1 is important for the regulation of T_FR_ suppressive function (31–36). Additionally, PD-1 and IL-2 signals are critical for T_FR_ cells. Mice deficient in PD-1 or its ligand PD-L1 have increased T_FR_ cell abundance with enhanced suppressive activity (37), while high IL-2 concentrations at the peak of influenza infection prevent T_FR_ cell development (38). However, the maintenance of developed T_FR_ cell stability appears to require the IL-2 signaling that is regulated by Blimp1 (34).

While T_FR_ cells are capable of regulating a variety of immune responses similar to conventional Tregs, they are uniquely known for their ability to regulate GC response and antibody production (27–30). Despite the low frequency, the importance of T_FR_ cells has been re-emphasized in a recent study in which a mouse model with a selective depletion of T_FR_ cells displays a profound alteration of immune responses, including increased self-reactive antibody (39). Several mechanisms for T_FR_-mediated suppression have been reported, including the one mediated by CTLA-4. Genetic deletion or blockade of CTLA-4 impairs T_FR_ cell development and function, leading to spontaneous T_FH_ differentiation and GC expansion (40, 41). T_FR_ cells are also shown to inhibit specific effector molecules, central metabolic and anabolic pathways in both T_FH_ and GC B-cells, but retain their transcriptional signature (42). This type of suppression appears durable and persists in their absence, and can be overcome by IL-21 signals (42). However, it remains unclear if T_FR_ cells directly target T_FH_ and/or B-cells during GC responses, and whether T_FR_ cells can regulate memory B-cells or plasma cells directly.

## Treg/T_FR_ Stability

Tregs must maintain their anergic phenotype and suppressive activity during ongoing inflammatory responses (43–45). This functional stability reflects a lack of effector activity by Tregs (i.e., expression of pro-inflammatory cytokines) and may or may not require maintenance of Foxp3 expression (44–46). Loss of Foxp3 (even a slight reduction) often results in the generation of ex-Tregs (47), while conversion into effector T-cells with unaltered Foxp3 expression is referred as Treg “fragility” (48). Several factors appear to be important for Treg stability/fragility, including CD25/STAT5 signals (43), PTEN/Akt/Foxo1/3a pathway (49–51), CARMA1–BCL10–MALT1 (CBM) signalosome complex (52), autophagy (53), Ezh2 (54, 55), Helios (56), Eos (57) and Nrp1 (48, 58). While the former 6 pathways regulate Foxp3, ablation of the latter 2 factors does not affect Foxp3 expression. Many of these pathways implicated in the context of tumor will be discussed in *Treg/T_FR_ Stability in the TME*. Here we focus on the CD25/STAT5/Foxp3-dependent regulation of Treg stability and function.

### Foxp3-Dependent Treg Stability

Foxp3 is crucial for maintaining Treg identity. Loss of Foxp3 results in Treg instability, dysfunction, and potential life-threatening autoimmune diseases (59–62). At steady state, Foxp3 expression and tTregs are incredibly stable (63). However, Tregs often become unstable under inflammatory conditions. Treatment of Tregs *in vitro* with proinflammatory cytokines like IL-4 and IL-6 results in the downregulation of Foxp3 and the upregulation of effector cytokines such as IFNγ (43, 64). Adoptive transfer of Foxp3^+^ Tregs into lymphodepleted mice also results in the loss of Foxp3 expression by a substantial population of Tregs, which appears to be limited to the CD25^lo^Foxp3^+^ subset as the majority of CD25^hi^Foxp3^+^ cells retain Foxp3 expression (65–67). While a portion of the Foxp3^–^ population, ex-Tregs, acquires Teff function, others are capable of reacquiring Foxp3 expression upon activation (66), suggesting the heterogeneity of Tregs and their ability to accommodate their function by adapting to environmental stimuli. These ex-Tregs are consistently reported to be autoreactive and pathogenic, causing autoimmune diseases upon adoptive transfer (35, 67–69).

### Mechanisms for Foxp3-Dependent Treg Stability

Mechanisms to reinforce Foxp3 expression and Treg stability have been extensively studied. TCR stimulation, along with the recruitment of transcription factors, such as NFAT, Foxo1 and Foxo3, to the *Foxp3* promoter, is the primary step in triggering *Foxp3* gene transcription (70–73). Additionally, the conserved non-coding sequence (CNS) elements at the *Foxp3* locus are important for Treg fate determination and lineage stability (74–76). The pioneer element CNS3 facilitates Foxp3 induction and increases the generation of both tTregs and pTregs. While tTregs do not rely on CNS1 for Foxp3 induction, CNS1 is indispensable for pTreg generation as it contains a TGF-β-NFAT response element and is dependent of TGF-β signaling to induce histone acetylation in the *Foxp3* enhancer region (76–78). CNS2, which contains the Treg specific demethylation region (TSDR), is crucial for the maintenance of Foxp3 expression in dividing Tregs (43, 76). CNS2, the CpG-rich region, is fully methylated in conventional T-cells, but largely demethylated in tTregs and partially methylated in pTregs. Upon TSDR demethylation, Foxp3, along with STAT5, NFAT and Cbfβ-Runx1, binds to CNS2, stabilizing Foxp3 expression through positive feedback mechanisms (62, 79–83). The availability of IL-2 and activation status of CD25/STAT5 signals that are modulated by several factors, including Helios and Blimp1 (34, 56), are essential for CNS2 to sustain Foxp3 expression, preventing Treg differentiation into Teff by counteracting proinflammatory cytokine signaling (43), which explains why CD25^hi^Foxp3^+^ cells are more stable than CD25^lo^Foxp3^+^ cells.

### Blimp1-Mediated Regulation of Treg/T_FR_ Stability

eTregs are marked by the expression of Blimp1 (10), however, its role in eTregs have been largely restricted to its regulation of IL-10 expression until recent findings from our group and others showing that it is important for Treg lineage stability and suppressive activity (34, 35). Consistent with the finding that expression of Blimp1 in the thymus is very low and Blimp1 unlikely regulates early T-cell development (84), mice with a Treg-specific deletion of Blimp1 do not show overt autoimmune phenotype (34, 35). However, Tregs from these mice are unstable with reduced Foxp3 expression and produce inflammatory cytokines after immunization, and these mice develop severe experimental autoimmune encephalitis (EAE) (34, 35, 68). At the peak of EAE, the presence of IL-6 activates the DNA methylating enzyme Dnmt3a, resulting in CNS2 methylation. Blimp1 is able to inhibit Dnmt3a upregulation and CNS2 methylation, thereby preventing the acquisition of a Teff phenotype (35). Additionally, Blimp1 can repress IL-23R-STAT3 signaling while retaining the CD25-STAT5 pathway in eTregs to sustain Foxp3 expression (34). Blimp1 is also critical for both T_FR_ lineage stability and their proper entry into the GC (34). Blimp1-deficient T_FR_ cells display an impaired suppressive phenotype *in vivo* with reduced Foxp3 and CTLA-4 expression, while increasing proinflammatory cytokines like IL-17A and IFNγ. These unstable T_FR_ cells prematurely migrate into the GC and differentiate into T_FH_-like cells, resulting in T_FH_ and GC B-cell expansion along with increased antibody and autoantibody production. Furthermore, adoptive transfer of Blimp1-deficient T_FR_ cells can promote pathogenesis associated with dysregulated GC responses (34, 68). Taken together, these studies have revealed Blimp1 as a new and central regulator of eTreg and T_FR_ lineage stability and suppressive capacity.

## Treg/T_FR_ Stability in the TME

Tregs are often recruited to the tumor microenvironment (TME) *via* various chemokines, such as CCL20, where they become highly activated and suppressive (11–13, 19, 85–87). Many pathways have been implicated in the regulation of TIL Treg stability.

### Pathways to Regulate Foxp3-Dependent TIL Treg Stability

A significant portion of TIL Tregs express PTEN and Foxo3a. The PTEN/Akt/Foxo3a pathway is important for the suppression of responses to apoptotic cells, including apoptotic tumor cells (49). Disruption of the PTEN/Akt/Foxo3a pathway through inhibition of PTEN results in Treg instability and the transitioning of suppressive Foxp3^+^ Tregs to proinflammatory ex-Tregs, leading to a more immunogenic microenvironment and substantial tumor regression (49–51). Disruption of the CBM signalosome complex also results in the acquisition of an anti-tumor effector phenotype by TIL Tregs, i.e., production of IFNγ, and reduced tumor growth. Increased IFNγ activates macrophages and upregulates PD-L1 by tumor cells. Accordingly, PD-1 blockade therapy along with CARMA-1 or MALT1 disruption eradicates tumors that do not respond to anti-PD-1 monotherapy, suggesting that induction of Treg instability confers the sensitivity to checkpoint inhibitor (52). Similarly, disruption of Ezh2 activity or depletion of Helios in Tregs leads to Foxp3 instability with an increased expression of effector cytokines like IFNγ and TNFα, enhanced anti-tumor immunity, and decreased tumor growth and progression (54, 55, 88). Importantly, colorectal cancers with abundant infiltration of FOXP3^lo^ non-suppressive T-cells display better prognosis than those infiltrated mainly with FOXP3^hi^ Tregs (18).

### Pathways to Regulate Foxp3-Independent TIL Treg Stability

Tregs can become unstable with an intact Foxp3 expression. The transcription factor Eos functions as a Foxp3 co-repressor to inhibit downstream target genes and to maintain Treg suppressive phenotype (89). In response to proinflammatory cytokines like IL-6, Eos but not Foxp3 is downregulated, leading to Treg reprogramming and the acquisition of a T_H_ phenotype with the upregulation of CD40L, IL-2, and IL-17A (57, 90). Co-transfer of “Eos-labile” Tregs results in more robust anti-tumor responses and better tumor control compared to transfer of Eos-stable Tregs. Moreover, reprogrammed Tregs upregulate CD40L and are able to facilitate DC cross-presentation to activate CD8^+^ T-cell anti-tumor response after vaccination with an tumor antigen (91).The Nrp1-Sema4a pathway is another mechanism for reinforcing TIL Treg function and limiting anti-tumor immune responses, while it is dispensable for the suppression of autoimmunity and the maintenance of immune homeostasis by Tregs. Ligation of Nrp1 on Tregs by Sema4a increases Treg survival and potentiates stable suppression with the increased production of IL-10 and IL-35, due to diminished Akt activation *via* the recruitment of PTEN (58, 92). Interestingly, loss of Nrp1 in Tregs results in high expression of IFNγ that drives the instability of surrounding wild-type Tregs. Consequently, mice with Nrp1-deficient Tregs display enhanced anti-tumor immunity and tumor clearance, prolonged survival and increased responsiveness to anti-PD-1 therapy without autoimmune abnormalities (48).

### Metabolic Pathways to Regulate TIL Treg Stability

Unlike Teff, Tregs favor oxidative phosphorylation but keep glycolysis under strict control, which plays an important role in shaping Treg identity and function (93, 94). The TME creates a low-glucose and high lactate environment that often promotes Treg suppressive function (95–99). Tregs may couple the survival mechanism, like autophagy to metabolic homeostasis by limiting glycolysis and reducing PI3K/Akt/Myc activation to ensure their integrity in the hostile TME (53). A most recent study has further elucidated that high-glucose conditions impair the function and stability of Tregs (100). However interestingly, Tregs have evolved to benefit from the symbiosis with tumors by utilizing the glycolytic by-product lactic acid to proliferate and prevent the destabilization effects of high glucose. This alternative pathway appears to be exclusively important for the stability and suppressive identity of tumoral but not peripheral Tregs. Similarly, limiting lipid uptake or metabolism by genetic or pharmacologic inhibition of FABP5 disrupts mitochondrial respiration, but also enhances Treg suppression by increasing IL-10 expression, suggesting another layer of complexity for the regulation of TIL Tregs (101).

### New Pathways to Regulate TIL Treg and T_FR_ Stability

Our recent study has revealed the importance of Blimp1 in the regulation of eTreg/T_FR_ stability and suppressive function under immune and autoimmune conditions (34, 68). However, the specific impact of Blimp1^+^ eTregs on, and mechanisms of action within, tumors are not yet explored. Since a majority of TIL Tregs express Blimp1 in some tumor models (102), and Blimp1 is suggested to be used for outcome prediction of cancer patients (103), loss of Blimp1 in eTregs may reprogram these cells into Teff, and potentially lead to increased anti-tumor immunity and decreased tumor progression, although this awaits further investigation. Importantly, these effects are likely restricted to TIL Tregs, since Blimp1 is expressed at low levels by Tregs at steady state (24). Despite a few reports showing that T_FR_ cells are significantly increased in cancer patients compared to healthy controls (104, 105), their mechanisms of action in the tumor are unclear. The increased TIL T_FH_ and B-cells, as likely observed in mice with the Treg-specific deletion of Blimp1, and tertiary lymphoid structure formation are associated with favorable outcomes in certain types of cancer and better responses to immunotherapy (106–112). Thus, it is important to define the contribution of T_FR_ cells to tumor progression and the impact of Blimp1 on T_FR_ function in the tumor.

## Therapeutic Approaches Targeting Treg Stability

Current cancer immunotherapy, particularly checkpoint inhibitor and CAR T-cell transfer, have shown great promise in some types of cancer. However, the success rates remain suboptimal (113–115), and some of these approaches are complicated with systemic immune-related adverse effects (116–118). Since Tregs, particularly eTregs, are one of major suppressive immune components in many cancers, most of these approaches are complicated with negative outcomes from Tregs in addition to positive effects on anti-tumor effector cells. For example, IL-2 can potently activate both T-cells and nature killer cells, and is potentially applicable for tumor control. However, IL-2 has the propensity to amplify Tregs, representing a major barrier for IL-2-based cancer therapy. The next generation of IL-2 that specifically targets tumor and preferentially boosts CD8^+^ T-cell response without inducing Treg responses appears to be promising (119). Similarly, high PD-1 expression is deleterious to Treg and T_FR_ suppression; anti-PD-1 may promote CD8^+^ T-cell anti-tumor response while inducing potent Treg/T_FR_-mediated suppression (37, 120). Therefore, the PD-1 expression balance between Teff and Tregs can predict the clinical efficacy of PD-1 blockade therapy, and needs to be considered when anti-PD-1 or anti-PD-L1-based therapy is applied (121). Interestingly, another checkpoint inhibitor, CTLA-4 blockade, has been recently shown to drive Treg instability in glycolysis-low tumors (122), a new mechanism beyond the conventional role of anti-CTLA-4 therapy in inducing Treg depletion.

**Figure 1:**
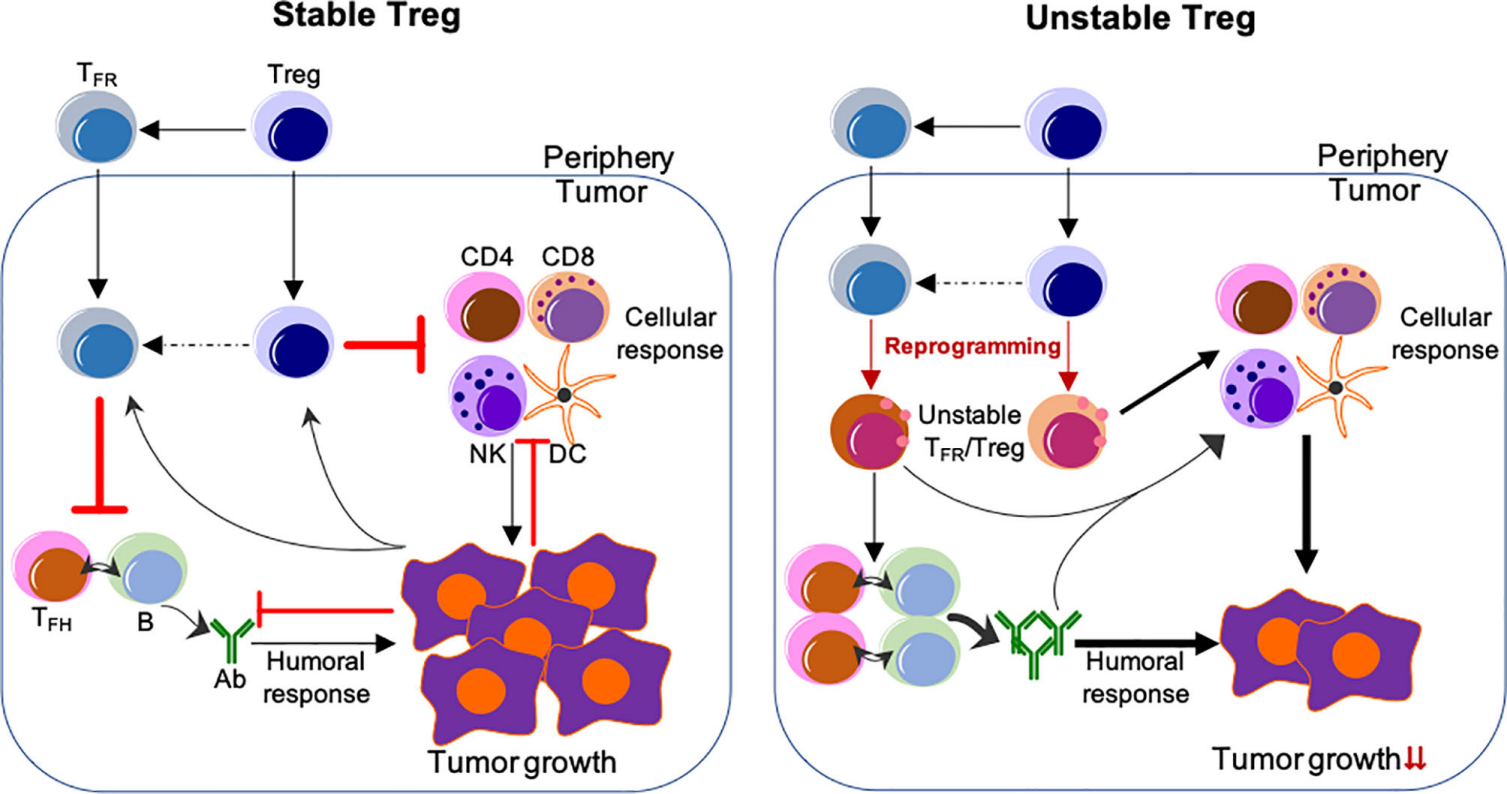
Reprogramming of TIL Tregs to control tumor by targeting their stability. *Left*, Stable Treg. Treg and T_FR_ cells mainly suppress the cellular and humoral anti-tumor immune responses, respectively. Conversely, tumor cells impose suppression on both cellular and humoral immune responses, but foster the immune suppression by Treg and T_FR_ cells. *Right*, Unstable Treg. Factors or approaches destabilize or reprogram Treg and T_FR_ cells into effector-like cells, which display impaired suppressive activity, but instead cooperate with both cellular and humoral anti-tumor components to control tumor growth and progression. The peripheral events are not depicted, but strategies used to selectively reprogram TIL Tregs, but not Tregs in the periphery, are expected to be most effective without systemic adverse effects. The unclear events are indicated by dashed lines. Not depicted: Peripheral T_FH_ and B-cells and their migration into the tumor; expansion of Treg/T_FR_ cells and anti-tumor effector cells; other cells regulating anti-tumor responses (e.g., myeloid-derived suppressor cells and macrophages, etc.).

Depletion of Tregs has been demonstrated to enhance anti-tumor responses, however, this ablation also results in lethal autoimmunity (60–62, 123). Studies from us and others suggest that a more effective approach would entail the specific reprogramming of TIL Tregs and reshaping the TME by employing the features of Treg instability, while not altering the stability of Tregs in the periphery (44, 45) (**Figure 1**). Disruption of the CBM signalosome complex or targeting Helios or Nrp1 or ligation of GITR in Tregs is shown to be effective for tumor control without peripheral autoimmune effects reported (48, 52, 88, 124). Based on the profound effect of Blimp1 depletion on the stability and suppressive ability of eTreg and T_FR_ cells, our findings suggest that targeting Blimp1^+^ eTreg may generate similar anti-tumor effects while limiting systemic toxicity. In addition to inducing eTreg destabilization (34), targeting Blimp1^+^ eTregs may also induce potent anti-tumor humoral responses, thus achieving multifaceted anti-tumor effects.

## Conclusion/Perspective

It is important to recognize that Treg stability can be manipulated to induce changes of immune responses, achieving the therapeutic benefit. Notably, loss of TIL eTreg stability in various tumors leads to remodeling of the TME from a suppressive state to an effective anti-tumor state and decreased tumor progression. Current and future challenges include the ability to selectively induce these changes in specific subsets of Tregs and in the TME but not systemically. As the field of cancer immunology progresses, understanding factors that regulate Tregs specifically in the tumor, yet have limited impact on Tregs in the periphery, is highly desirable and important for treating nearly every cancer patient, particularly any patient treated with immunotherapy, as it will direct the development of effective, targeted immunotherapies with reduced adverse events. This represents a new direction for how to manipulate Treg activity for cancer treatment.

## Author Contributions

MLD, JDL, and JWL drafted the manuscript and revised it critically. All authors contributed to the article and approved the submitted version.

## Conflict of Interest

The authors declare that the research was conducted in the absence of any commercial or financial relationships that could be construed as a potential conflict of interest.

## Publisher’s Note

All claims expressed in this article are solely those of the authors and do not necessarily represent those of their affiliated organizations, or those of the publisher, the editors and the reviewers. Any product that may be evaluated in this article, or claim that may be made by its manufacturer, is not guaranteed or endorsed by the publisher.

## Glossary

**Table:**

Bcl6	B-cell lymphoma 6 protein
BCL10	B-cell lymphoma/leukemia 10
Blimp1	B lymphocyte-induced maturation protein 1
CAR	chimeric antigen receptor
CARMA1	caspase recruitment domain-containing membrane-associated guanylate kinase protein-1
Cbfβ	core-binding factor subunit beta
CBM	CARMA1–BCL10–MALT1
CD40L	CD40 ligand
CNS	conserved non-coding sequence
CCR7	CC receptor 7
CTLA-4	cytotoxic T lymphocyte antigen 4
cTreg	central Treg
CXCR5	C-X-C chemokine rector 5
CXCL13	C-X-C chemokine ligand 13
Dnmt3a	DNA (cytosine-5)-methyltransferase 3a
EAE	experimental autoimmune encephalitis
eTreg	effector Treg
Ezh2	enhancer of zeste homolog 2
FABP5	fatty acid binding protein 5
Foxo3	forkhead box O3
Foxp3	forkhead box protein P3
GC	germinal center
GITR	glucocorticoid-Induced tumor necrosis factor receptor
ICOS	inducible T cell costimulatory
IFN	interferon
IL	interleukin
IL23R	IL-23 receptor
IRF4	interferon regulatory factor 4
MALT1	mucosa-associated lymphoid tissue lymphoma translocation protein 1
mTOR	mechanistic target of rapamycin
NFAT	nuclear factor of activated T-cells
Nrp1	neuropilin-1
PD-1	programmed death 1
PD-L1	programmed death ligand 1
PI3K	phosphoinositide 3-kinase
PTEN	phosphatase and tensin homolog
pTreg	peripheral Treg
Runx1	runt-related transcription factor 1
Sema4a	semaphorin 4a
STAT	signal transducer and activator of transcription
TCR	T-cell antigen receptor
Teff	effector T-cells
T_FH_	follicular helper T
T_FR_	follicular regulatory T
TGF-β	transforming growth factor β
T_H_	T helper
TIL	tumor-infiltrating lymphocytes
TME	tumor microenvironment
TNF	tumor necrosis factor
Treg	regulatory T-cells
TSDR	Treg specific demethylation region
tTreg	thymic Treg
